# Flexion bonding transfer of multilayered graphene as a top electrode in transparent organic light-emitting diodes

**DOI:** 10.1038/srep17748

**Published:** 2015-12-02

**Authors:** Jong Tae Lim, Hyunkoo Lee, Hyunsu Cho, Byoung-Hwa Kwon, Nam Sung Cho, Bong Kuk Lee, Jonghyurk Park, Jaesu Kim, Jun-Han Han, Jong-Heon Yang, Byoung-Gon Yu, Chi-Sun Hwang, Seong Chu Lim, Jeong-Ik Lee

**Affiliations:** 1 Soft I/O Interface Research Section, Electronics and Telecommunications Research Institute, Daejeon 305-700, Korea; 2 IT Convergence Technology Research Laboratory, Electronics and Telecommunications Research Institute, Daejeon 305-700, Korea; 3 Smart I/O Control Research Section, Electronics and Telecommunications Research Institute, Daejeon 305-700, Korea; 4 Department of Energy Science, Sungkyunkwan University, Suwon 440-746, Korea; 5 IBS Center for Integrated Nanostructure Physics, Institute for Basic Science (IBS), Sungkyunkwan University, Suwon 440-746, Korea

## Abstract

Graphene has attracted considerable attention as a next-generation transparent conducting electrode, because of its high electrical conductivity and optical transparency. Various optoelectronic devices comprising graphene as a bottom electrode, such as organic light-emitting diodes (OLEDs), organic photovoltaics, quantum-dot LEDs, and light-emitting electrochemical cells, have recently been reported. However, performance of optoelectronic devices using graphene as top electrodes is limited, because the lamination process through which graphene is positioned as the top layer of these conventional OLEDs is a lack of control in the surface roughness, the gapless contact, and the flexion bonding between graphene and organic layer of the device. Here, a multilayered graphene (MLG) as a top electrode is successfully implanted, via dry bonding, onto the top organic layer of transparent OLED (TOLED) with flexion patterns. The performance of the TOLED with MLG electrode is comparable to that of a conventional TOLED with a semi-transparent thin-Ag top electrode, because the MLG electrode makes a contact with the TOLED with no residue. In addition, we successfully fabricate a large-size transparent segment panel using the developed MLG electrode. Therefore, we believe that the flexion bonding technology presented in this work is applicable to various optoelectronic devices.

Organic light-emitting diodes (OLEDs) have attracted considerable attention as next-generation display and lighting devices, because of their light weight, high flexibility, low thickness, and low electric power consumption^1^,^2^,^3^,^4^. Transparent OLEDs (TOLEDs) are also candidates for future displays and lighting sources, because they have the potential for use in diverse applications such as smart windows, retail advertising, illumination, and head-up displays on car windshields^5^,^6^,^7^. In TOLEDs, the transparent conducting top electrode (TCTE) is one of the most essential parts that directly determines the device performance. This is because TOLEDs emit light from both the top and bottom sides of the device. A wide variety of materials and processes can be used in the bottom electrode; however, the top electrode choices are limited, because this material must be deposited on the OLED organic layer directly. Therefore, efficient TCTEs in OLEDs should have high optical transmittance and electrical conductivity, and they should not damage the organic layers during the deposition process. Moreover, it is necessary to obtain the appropriate energy level alignment between the organic layer and TCTE in order to facilitate efficient charge injection^8^.

TCTEs composed of a wide variety of materials have been developed, including semi-transparent metals (STMs)^5^,^9^,^10^,^11^, transparent conducting oxides (TCOs)^12^,^13^, conducting polymers^14^,^15^, carbon nanotubes (CNTs)^16^,^17^,^18^, silver nanowires (AgNWs)^19^, and graphene^8^,^20^,^21^. In conventional TOLEDs, TCOs and STMs are the materials of choice for commercial use, as the bottom and top electrodes, respectively. However, the use of STMs as top electrodes in OLEDs causes considerable light loss during the light out-coupling process. This is because STMs have strong absorption and a strong surface plasmon polariton (SPP) mode at the interface between the top organic layer and the TCTE^22^. Furthermore, the high reflectance of STMs causes color degradation, through the generation of undesirable noise peaks and the distortion of the electroluminescence (EL) spectrum, depending on the viewing angle^23^. In the case of TCOs, such as an indium tin oxide (ITO) and indium zinc oxide (IZO), plasma from the deposition process can cause undesirable degradation of the organic materials in the OLEDs^24^. Recently, TCTEs composed of conducting polymer^14^,^15^, CNTs^16^,^17^,^18^, and AgNWs^19^, are realized using solution process. However, the process can cause the damage and generation of pin holes at the interface.

Graphene is a good candidate for use in TCTEs, because it exhibits uniform optical transmission in a visible spectral range, while also exhibiting good electrical conductivity, a weak SPP mode, good stretchability, and excellent chemical stability in air and acidic environments^25^,^26^,^27^. However, it is difficult to deposit graphene onto organic layers directly, because of the high required synthesis temperature^25^. Previously reported graphene top electrodes in light-emitting devices have been prepared using wet transfer method or high-temperature thermal treatment^8^,^20^. These graphene transfer processes limit the materials and structures of these devices, leading to poor device performance. The adoption of graphene as a TCTE in conventional OLEDs is therefore associated with a number of challenges. First, the interface between the graphene TCTE and the organic layer must comprise a conformal contact that is free from air gaps, so as to facilitate homogenous charge injection. Second, a residue-free graphene transfer process is imperative, because the light-emitting performance can be undermined by contaminants at the interface. Third, the graphene TCTE must be transferred to the flexion surface structure, because the pixels in a practical OLED display are commonly defined based on the bank structure, via a height gradation. The high bending stress of the graphene TCTE typically results in poor device performance, due to the occurrence of voids in the bonding process. Therefore, the graphene TCTE supporting layer must have a low Young’s modulus, so that it is flexible enough to tightly contact to the flexion patterns^20^,^28^,^29^. Finally, the graphene TCTE transfer process for OLED fabrication must be done at a low-temperature and dry procedure, with no out-gassing.

In this study, we introduce an elastic graphene bonding structure (EGBS) comprised of a residual-free large-area multilayered graphene (MLG)/bonding layer (BL)/polyethylene terephthalate (PET) structure as a TCTE. We also demonstrate a dry laminating technology for the fabrication of a top graphene-electrode-based TOLED (G-TOLED), which enables uninterrupted contact by impurities and bending stress between the MLG and the top organic layer with the flexion bank patterns. Optical imaging, atomic force microscopy (AFM), and polarized Raman spectroscopy analyses indicate the presence of high-quality, smooth-surface, residue-free graphene in the bonding structure. Additionally, in terms of optical and electrical device characteristics, the G-TOLED exhibits superior performance to a thin-Ag top-electrode-based TOLED (Ag-TOLED).

## Results

### EGBS fabrication process

**Figure 1:**
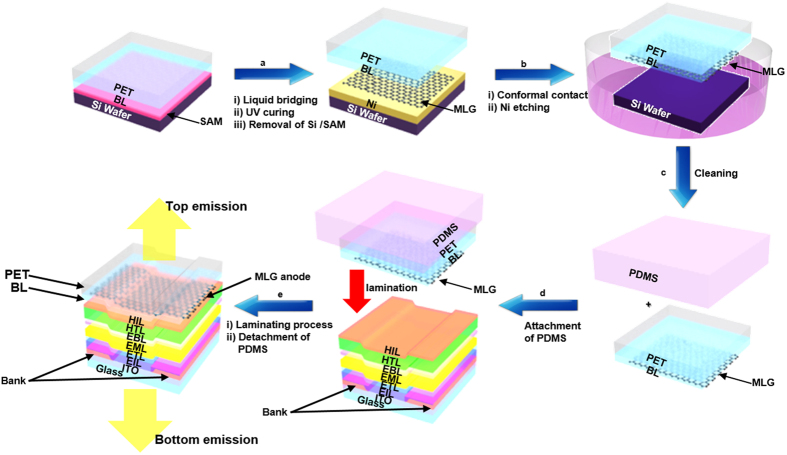
Overall fabrication process of the G-TOLEDs. **(a)** Liquid bridging process, UV curing, and removal of the Si WF/SAM for fabricating a BL/PET sample, **(b**) processes of conformal contact and Ni etching for fabricating a MLG/BL/PET bonding structure, **(c)** cleaning process of a MLG/BL/PET sample, **(d)** fabrication process of a MLG/BL/PET/PDMS sample, and **(e)** lamination process for the G-TOLED fabrication.

Figure 1 shows a schematic diagram of the process used to fabricate and transfer the EGBS onto an OLED stack, consisting of glass/ITO/organics, so as to create the G-TOLED. In the EGBS, the PET is employed as a supporting layer. There are three essential steps in this process: 1. Control of the surface roughness of the BL/PET structure (Fig. 1a); 2. Transfer of the MLG onto the BL/PET (Fig. 1b,c); and 3. Fabrication of a G-TOLED by laminating the EGBS on the glass/ITO/organics stack (Fig. 1d,e) (see the Methods section for more details).

Figure 1a shows the first step, in which the BL roughness is controlled. A silicon wafer (Si WF) with a surface roughness of 0.23 nm was used as the BL mould (see Supplementary Information S1 and S2 online for details), and treated with a self-assembly monolayer (SAM) to create lower surface energy on the Si WF/SAM. This facilitated easy separation of the BL/PET from the Si WF/SAM/BL/PET. Here, the bonding layer was formed via ultraviolet (UV) curing of a liquid-type bonding agent that bridged the Si WF/SAM and the PET film (see the Methods section for details). Then, the BL/PET sample was detached from the Si WF/SAM/BL/PET sample. Thus, the SAM-treated Si WF shown in Fig. 1a was incorporated to obtain a smooth BL surface.

As shown in Fig. 1b, a MLG/BL/PET structure was fabricated by establishing a contact between the Si WF/Ni/MLG and the BL/PET sample; this was followed by nickel (Ni) etching. The BL of DMS-R22 is expected to have a similar Young’s modulus to polydimethylsiloxane (PDMS), with approximately 0.6–8.2 MPa^29^. This low Young’s modulus allows an adhesion without air gaps to be formed between the MLG and BL (see the Methods section). The adhesion of the MLG to the BL was not disturbed during the etching of the Ni layer, which was conducted using a ferric chloride (FeCl_3_) solution. Additionally, during the cleaning step shown in Fig. 1c, the backside of the MLG on the BL/PET becomes free of particles because Ni and Fe complexes that remain on the etched face can be easily removed by washing in water and a dilute hydrochloric solution after the Ni etching process. The EGBS was dried at 60 °C under vacuum.

Finally, Fig. 1d,e show the attachment of the PDMS to the EGBS, the lamination of the graphene bonding structure on the OLED stack, and the separation of the PDMS. During the lamination, the PDMS homogenizes the applied force on the structure, preventing the device from becoming locally damaged by the pressure. The lamination was proceeded at a low temperature of 60 °C and at a low pressure of approximately 110 kPa. Also, the EGBS and the underlying OLED stack were held in place by the dispersive adhesion between the BL and the adjacent organic layer^28^. This mild adhesion prevented the organic layers from being damaged during the process.

### EGBS characterization

**Figure 2:**
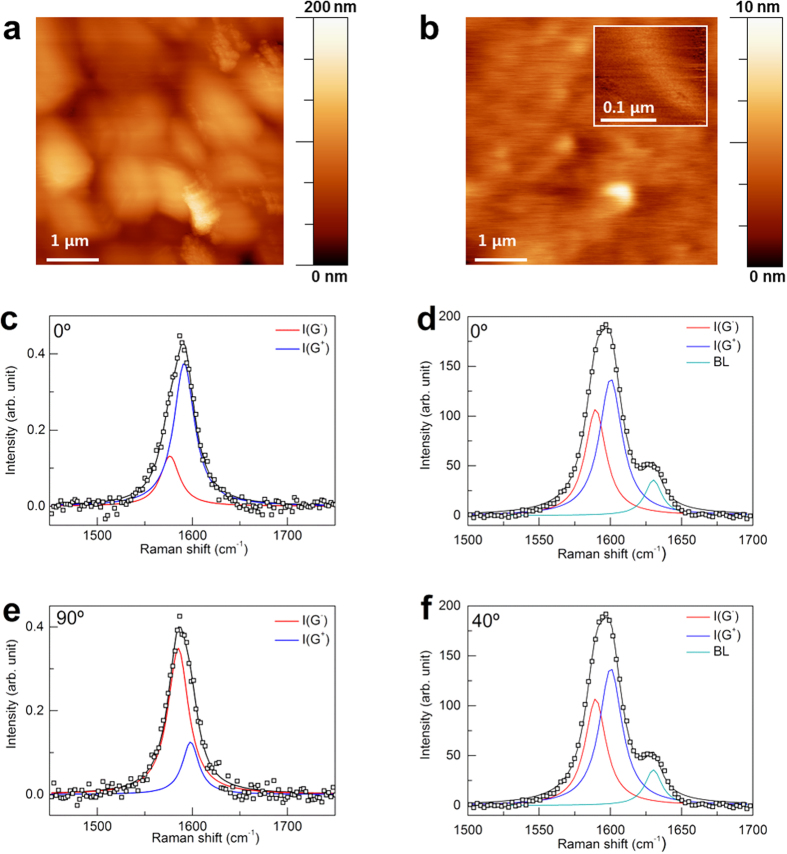
Atomic force microscopic images and polarized Raman spectra of EGBS. Microscopy of **(a)** Si WF/Ni/MLG and **(b**) MLG/BL/PET measured over an area of 5 × 5 μm^2^. The inset shows the RMS roughness of BL/PET measured over an area of 0.2 × 0.2 μm^2^. The maximum value of the height scale bars are 200 nm **(a)** and 10 nm **(b)**, and 10 nm (the inset of **(b)**). The intensity variation of G^−^ and G^+^ peaks of Si WF/Ni/MLG at **(c)** 0° and **(e)** 90° of polarization angle of the incident laser. The intensity variation of G^−^ and G^+^ peaks of MLG/BL/PET at **(d)** 0° and **(f)** 40° of polarization angle of the incident laser.

The AFM images of the Si WF/Ni/MLG and the EGBS shown in Fig. 2a,b were obtained over 5 × 5 μm^2^ using the tapping mode. The root-mean-square (RMS) surface roughness of the Si WF/Ni/MLG was approximately 24 nm, which may have been due to numerous grains with different growth rates and their boundaries. The flattened surface of the MLG was achieved via our unique transfer process, in which Si WF/SAM and BL/PET were employed, as shown in Fig. 1. During this process, the MLG was separated from the Si WF/Ni/MLG and the MLG of the Ni side (backside MLG) was used to create a conformal contact with the organic layer of the OLED stack. Therefore, the surface roughness of the EGBS decreased to approximately 0.88 nm, which is 30 times smoother than that of the Si WF/Ni/MLG (front side). The surface roughness values of the various components used in the EGBS fabrication process were 0.23 nm for the Si surface, 0.36 nm for the Si WF/SAM (see Supplementary Information S2 online), 0.52 nm for the BL/PET, and 0.88 nm for the EGBS (as shown in Fig. 2b, inset). This indicates that the smoothness of the Si wafer was reproduced on the BL/PET through this process. Consequently, our approach yields reduced MLG surface roughness compared to that of samples fabricated via a conventional transfer method, for which MLG surface roughness profiles with protrusions of several tens of nanometers and residues and ripple structures have been reported^30^.

The Raman spectra of the G band were also studied using a 532-nm laser, so as to assess the strain exerted on the MLG in the EGBS (see Supplementary Information Fig. S3 online). As a means of probing the local strain on the MLG, polarized Raman spectroscopy was performed. In this characterization, the intensity variations and peak positions of the G^+^ and G^−^ bands were studied as functions of the polarization angles of an incident laser with respect to that of the detector. The polarization angle was controlled using a half-wave plate to vary the polarization direction of the incident light. The G band was chosen as a measure of the local strain, because it is not energy dispersive, unlike the 2D band. Although the G band is sensitive to doping, the doping effect has no polarization dependence. Therefore, the local strain experienced by the MLG can be considered to be laser-energy independent.

Figure 2c–f, are the G-band peaks of the Si WF/Ni/MLG and MLG/BL/PET, respectively. Without strain, no peak intensity variations in response to varying incident-light polarization angle were observed. As the strain grew, the crystal symmetry broke; this contributed to the separation of the G band into G^+^ and G^−^ sub peaks. As shown in Fig. 2c,e, the G^+^ and G^−^ peak intensities vary significantly with respect to the polarization angle. This indicates that the MLG on the Ni experienced a strain. The intensity plots of the G^+^ and G^−^ bands as functions of the polarization angle do not exhibit sinusoidal behavior (see Supplementary Information Fig. S3a and S3b online), which appears under uniaxial strain. Thus, it is suggested that both compressive and tensile strains coexist in random directions on CVD-grown MLG. The existence of this strain is attributed to the polycrystalline nature of the MLG, on which graphene grains with various crystalline directions are stitched through the grain boundaries. A mismatch in the growth direction and the number of layers in each grain causes uncollimated strain. Therefore, we averaged the peak positions of the G^+^ and G^−^ band from 0 to 90°, in order to estimate the local strain. The averaged G^+^- and G^−^-band peak positions were 1593.5 and 1580.5 cm^−1^, respectively. Then, the local strain was estimated from the expression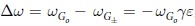, where 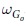
is the G-band peak of unstrained graphene, γ = 2 is the Gruneisen parameter^31^,^32^, and *ε* is Young’s modulus. Our estimation indicated that the strain level of the Si WF/Ni/MLG was G^+^ = 4.2 GPa and G^−^ = 0.017 GPa. Note that the upshift of the G-band peaks implies the presence of compressive strain on the Si WF/Ni/MLG.

Figure 2d,f show a deconvoluted G-band peak on the MLG/BL/PET, which reveals three substructures. In this case, a shoulder peak can be seen at 1630 cm^−1^, which originated from the BL/PET substrate (see Supplementary Information Fig. S3 online). The lack of angle dependence of the shoulder peak rules out the possibility of a D´ peak at 1630 cm^−1^. Similar to the Si WF/Ni/MLG, the strain on the MLG/BL/PET was determined to be multiaxial. Therefore, we averaged the peak positions of the G^+^ and G^−^ bands and extracted the local strain. The evaluated local strain on the MLG/BL/PET surface was found to be G^+^ = 6 GPa and G^−^ = 32 GPa. The averaged G^−^- and G^+^-band peak positions were therefore 1590 and 1600.3 cm^−1^, respectively (see Supplementary Information Fig. S3c and S3d online). As both the G^+^ and G^−^ bands shifted to higher energies, it is assumed that a significant compressive strain was applied on the MLG by the BL.

To investigate the metal complex traces following the Ni etching process, wide-scan X-ray photoemission spectroscopy (XPS) was conducted at a photon energy of 650 eV (see Supplementary Information S4 online). The EGBS spectrum did not indicate the presence of Cl 2p (199 eV), Cl 2s (271 eV), Ni 3p (67 eV), or Fe 3p (53 eV), which are related to the FeCl_3_-solution-based etching process. This clean interface indicates that no impurities were present on the MLG adhering to the organic layer of the OLED stack.

### Flexion transfer of EGBS

**Figure 3:**
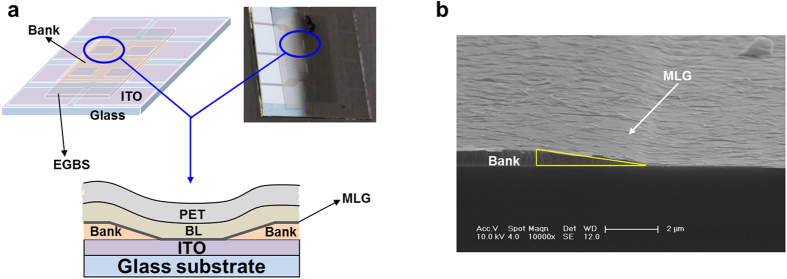
Flexion transfer of EGBS on an ITO glass. (**a**) Schematic planar image (left panel), an optical image (right panel), and schematic cross-sectional view (bottom panel) of a glass/ITO/EGBS with bank patterns. (**b**) Cross-section SEM image of MLG transferred on a glass/ITO with the bank patterns.

One of the instrumental device parameters of G-TOLEDs is the mechanical flexibility of the EGBS, because the EGBS is transferred to the bank structure of the OLED stack that is gradually recessed by 1 μm below the surface of the substrate. To confirm the flexibility of EGBS using scanning electron microscopy (SEM), EGBS is transferred on glass/ITO with bank patterns shown in Fig. 3a schematically (left panel) and optically (right panel). A cross-sectional view of a glass/ITO with bank patterns marked by a circle in both images is schematically shown at the bottom of Fig. 3a. Figure 3b is cross-section SEM image of a pixel with a bank structure, on which only MLG is transferred. The angle of the deflection point of the bank surface is 12°, which is marked by a triangle in Fig. 3b. The pixel size is 1.5 × 1.5 mm^2^. For uniform light emission, the EGBS must make tight contact with the corrugated surface of the OLED stacks, without gaps caused by the bending of the EGBS. To accommodate bending stress, the PET must be sufficiently flexible. Therefore, we evaluated the performance of various BL/PET thicknesses in this regard. We have confirmed that a PET film with less than 25-μm thickness closely followed the bank structure, with no stress-induced physical separation. In Fig. 3b, the corrugated features are a result from local charging and electron-beam heating on the surface of glass/ITO/MLG by electron beam.

### TOLED characterization

As schematically shown in Fig. 1e, we fabricated yellowish-green phosphorescent inverted TOLEDs with MLG top electrodes using glass (0.7 mm) as a substrate, ITO (70 nm) as a cathode, lithium (Li)-doped TRE (5%, 30 nm) as an electron-injecting layer (EIL), TRE (25 nm) as an electron-transporting layer (ETL), (1–6)Bis(2-phenylpyridinato)[2-(biphenyl-3-yl)pyridinato]iridium(III) (Ir(ppy)_2_(m-bppy))-doped PGH02 (8%, 20 nm) as an emitting layer (EML), 4,4′,4″-tris(N-carbazolyl)-triphenylamine (TcTa, 10 nm) as an electron-blocking layer (EBL), 1,1-bis((di-4-tolylamino)phenyl)cyclohexane (TAPC, 70 nm) as a hole-transporting layer (HTL), 1,4,5,8,9,11-hexaazatriphenylene-hexacarbonitrile (HAT-CN, 30 nm) as a hole-injecting layer (HIL), MLG as an anode (approximately 3 nm), BL, and PET (25 μm). We also fabricated a device with the same structure, but with a thin-Ag (20 nm) top electrode, for use as a reference (see the Methods section).

**Figure 4:**
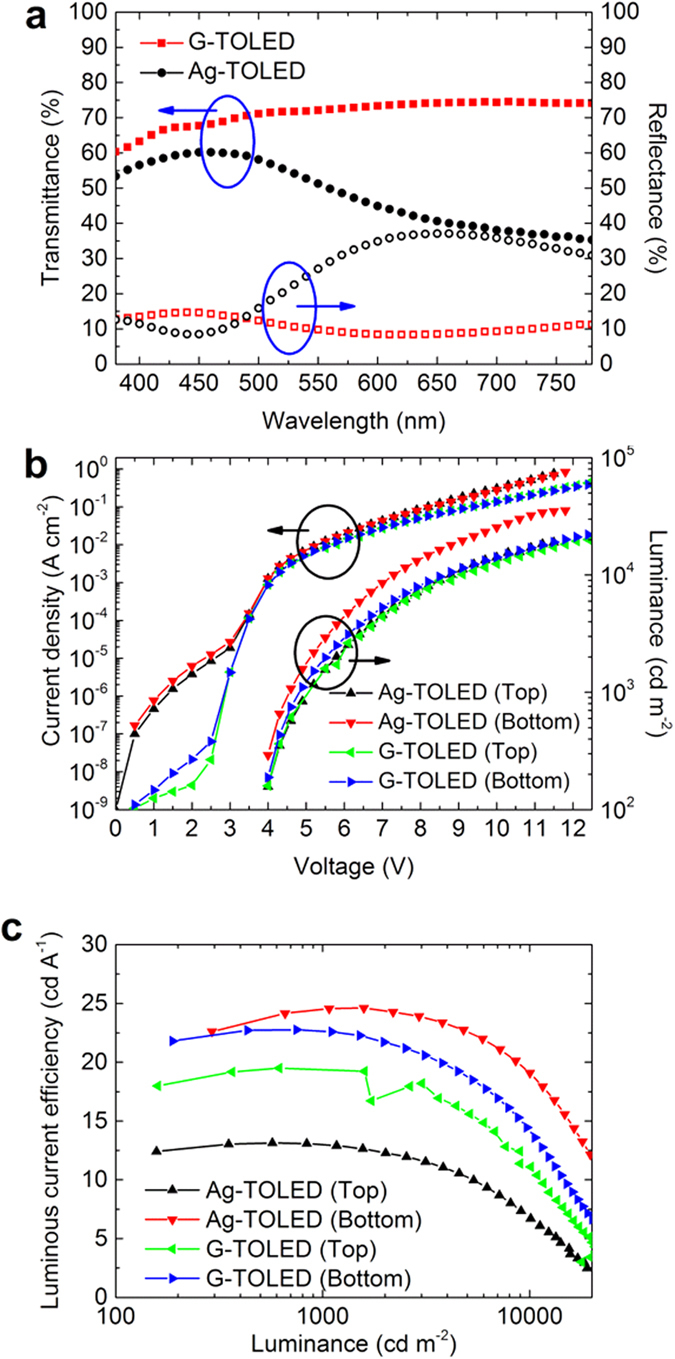
Device characteristics of the Ag-TOLED and G-TOLED. **(a)** Transmittance and reflectance spectra in visible spectral region, **(b)** current density-voltage vs. luminance-voltage curves, and **(c)** luminous current efficiency-luminance curves.

Figure 4a shows the transmittance and reflectance of the devices with the two different top electrodes, i.e., the G- and Ag-TOLEDs, in the visible spectral region. The former device exhibits higher transmittance and lower reflectance than the latter. For instance, the transmittance at 550 nm is approximately 72.1% for the G-TOLED but approximately 51.3% for the Ag-TOLED. Further, the reflectance of the G-TOLED is lower and more uniform than that of the Ag-TOLED, which is both high and non-uniform, especially above 500 nm. The reflectance at 550 nm is 9.8% for the G-TOLED, but approximately 27.1% for the Ag-TOLED. These results indicate that the top MLG electrode yields an advantage compared to the top Ag electrode in terms of optical performance. Figure 4b shows the current density-voltage-luminance (J-V-L) characteristics of the G- and Ag-TOLEDs. The J-V characteristics of each pixel in the individual devices are highly consistent, which contributes to the uniform top and bottom emission of both devices. The current density of the Ag-TOLED is similar to that of the G-TOLED in the voltage range of 4–6 V. However, the Ag-TOLED exhibits a slightly higher current density at higher voltage values compared with that of the G-TOLED; this is because the Ag sheet resistance is lower than that of MLG. However, both devices have almost the same turn-on voltages, implying that the energy barrier between the Ag and HIL is similar to that between the MLG and HIL. This is because the HAT-CN has the same capacity for electron extraction from the HTL, despite the work function difference between the Ag (approximately 4.3 eV) and MLG (approximately 4.6 eV)^33^,^34^,^35^.

The Ag-TOLED luminance at 4.6 V reaches 1,075 and 572 cd m^−2^ for the bottom and top emission, respectively. However, the G-TOLED luminance at the same voltage is 749 cd m^−2^ for the bottom emission and 617 cd m^−2^ for the top emission. The total luminance of the G-TOLED is comparable to that of the Ag-TOLED. In addition, total maximum luminance of G-TOLED is 44,293 cd m^−2^ (23,428 cd m^−2^ for bottom and 20,865 cd m^−2^ for top) at 13 V which is about 43 times higher compared to previously reported value^8^. These results suggest a successful lamination and subsequent uniform hole injection of the MLG top electrode. For the Ag-TOLED, the bottom-to-top emission ratio measured at 4.6 V is approximately 1.88, but that of the G-TOLED is approximately 1.21. Also, the emission ratios for both devices measured at 7.9 V are almost identical, at 1.84 and 1.16 for the Ag- and G-TOLED, respectively. The larger bottom-to-top emission ratio of the Ag-TOLED is due to the low transmittance and high reflectance of the Ag compared to the MLG, as shown in Fig. 4a. Therefore, the Ag-TOLED exhibits higher bottom-emission luminance compared to that of the top emission, whereas both sides of the G-TOLED exhibit relatively similar luminance.

Figure 4c shows the luminous current efficiency (LCE)-luminance characteristic curves of the devices fabricated with Ag and MLG top electrodes. The LCE of the Ag-TOLED is higher than that of the G-TOLED for the bottom emission, but it is considerably lower than that of the G-TOLED for top emission. For instance, the LCEs of the Ag-TOLED are 24.6 cd A^−1^ at 1075 cd m^−2^ for bottom emission and 13.1 cd A^−1^ at 572 cd m^−2^ for top emission, whereas the LCEs of the G-TOLED are 22.8 cd A^−1^ at 749 cd m^−2^ and 19.5 cd A^−1^ at 617 cd m^−2^ for bottom and top emission, respectively. In other words, the total LCEs of the devices fabricated with Ag and MLG are approximately 37.7 cd A^−1^ at 1647 cd m^−2^ and 42.3 cd A^−1^ at 1366 cd m^−2^, respectively. This result indicates that the total LCE of the G-TOLED outperforms that of the Ag-TOLED by approximately 12.2% for this device structure. It is shown that our flexion transfer method is highly reproducible, which was confirmed from the test with multiple number of devices (Supplementary Information Fig. S5 on line).

**Figure 5:**
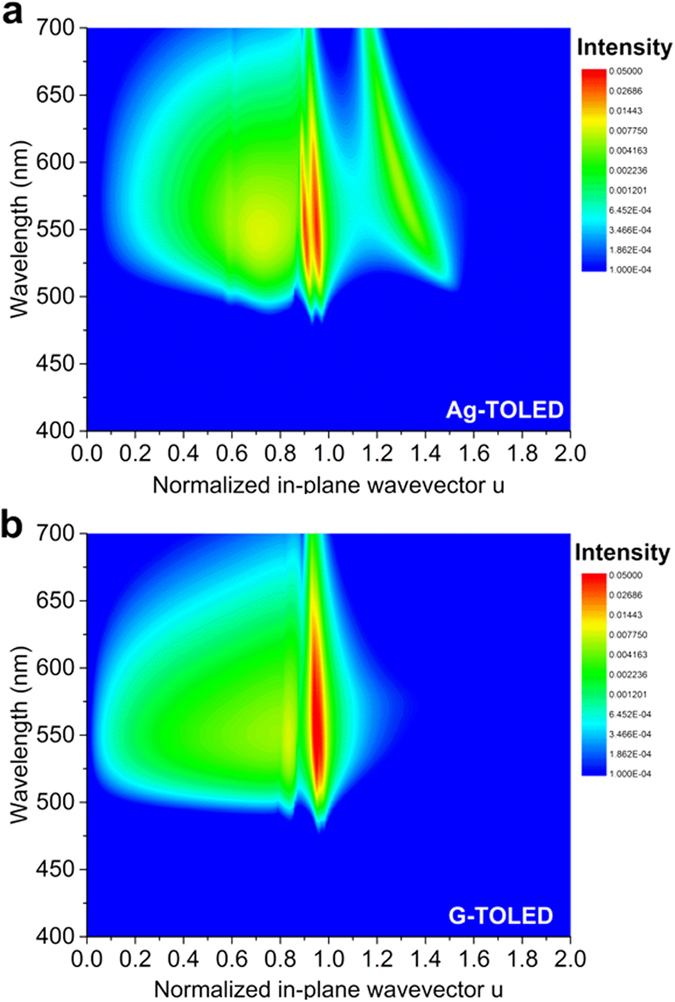
Simulation of the total dissipated optical power. (**a**) Ag-TOLED and (**b**) G-TOLED.

To investigate the optical efficiency of the G-TOLED in comparison with that of the Ag-TOLED, we simulated the power dissipation spectra using the classical dipole model^36^, as shown in Fig. 5a,b. It was assumed that the emitting molecules were isotropically oriented and that the intrinsic photoluminescence quantum yield was unity in this simulation. Here, the normalized in-plane wave vector (*u*) denotes the normalized transverse wave vector and is defined as *u* = *k*_*p*_*/k*, with *k* as the total wave vector at the emitter location and *k*_*p*_ as the projection of the wave vector in the source plane. If *u* > 1, the waves are universally evanescent. Then, the radiated power is transferred to non-radiative modes, such as SPPs, in this region due to the metal electrodes in the OLEDs^37^. The intensity of the power dissipation in the Ag-TOLED at the *u* value, which is greater than unity, is larger than that of the G-TOLED. The simulated result at 550 nm is depicted in Supplementary Information Fig. S6 (online). This result indicates that use of the Ag top electrode leads to a stronger SPP loss compared to that obtained with the MLG top electrode, because the large electron concentration in Ag results in an SPP mode in the visible range. In contrast, the low electron concentration of MLG causes electron plasmons to occur below the far infrared (IR) region, so no SPP occurs in the visible range^22^,^38^,^39^. Table 1 lists the detailed simulation results. The out-coupled efficiencies are 8.97% (bottom) and 3.06% (top) and 7.94% (bottom) and 7.83% (top) for the Ag-TOLED and G-TOLED, respectively. These values correspond well with the experimental values of 8.20% (bottom) at 922.6 cd m^−2^ and 2.85% (top) at 483.1 cd m^−2^ (total 1405.7 cd m^−2^), measured at approximately 4.5 × 10^−3^ A cm^−2^, and 6.60% (bottom) at 748.6 cd m^−2^ and 6.44% at 616.7 cd m^−2^ (total 1365.3 cd m^−2^), measured at approximately 3.2 × 10^−3^ A cm^−2^, for the devices with the Ag and MLG top electrodes, respectively. To determine the external quantum efficiencies (EQE) of the devices, we also measured the angular distributions of their EL intensities and their spectra, as shown in Supplementary Fig. S7 (online). The EQE is calculated using angle-resolved EL properties with interpolation, not lambertian assumption. The measured efficiencies were slightly lower than the simulated values, because the actual devices did not have an internal efficiency of unity as a result of electrical loss. These results suggest that the reduced SPP mode in the G-TOLED does not fully convert to the air mode, but the other modes in the G-TOLED are re-distributed. Therefore, the air mode increases slightly compared with that of the Ag-TOLED. In addition, reduction of the SPP mode can render the realization of higher efficiency OLEDs possible, through the adoption of a number of other light out-coupling techniques.

**Figure 6:**
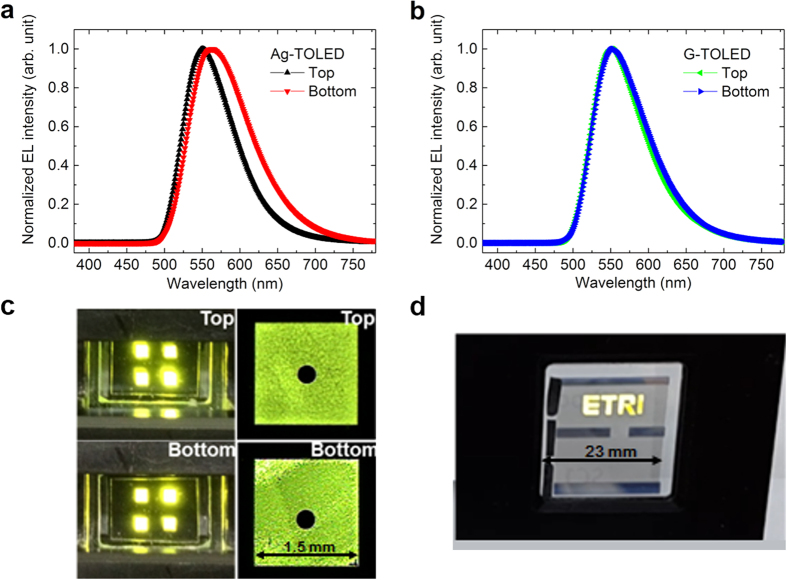
EL Spectra and photographs of TOLEDs. Normalized top and bottom EL spectra of **(a)** the Ag-TOLED and **(b**) the G-TOLED, **(c)** top and bottom pixel images during operation with active size of 1.5 × 1.5 mm^2^ of a G-TOLED, **(d)** “ETRI” logo displayed from an OLED segment panel with an MLG top electrode (the area of MLG is 23 × 23 mm^2^).

Figure 6a,b show the normalized EL spectra of the devices fabricated with Ag and MLG top electrodes measured at 4.6 V. The top-side EL spectrum of the Ag-TOLED differs from that of the bottom-side EL spectrum. The peak wavelength and the full width at half maximum (FWHM) of the top-side EL spectrum are 551 and 78 nm, respectively, but those of the bottom-side are 563 and 94 nm, respectively. However, the G-TOLED exhibits peak wavelengths and FWHMs of 552 and 81 nm for the top emission and 553 and 84 nm for the bottom-emission, respectively. The MLG top electrode affects the EL spectrum of the EML only slightly compared to the thin-Ag top electrode, because the former has a considerably lower optical reflectance, resulting in low optical interference^40^. This finding suggests that the EL spectrum is not distorted by the MLG top electrode. Figure 6c shows images of the yellowish-green top- and bottom-emitting pixels of the G-TOLED. Supplementary Video 1 that features the operation of G-TOLED is provided. The magnified pixel images show that the MLG top electrodes were laminated in conformation with the organic layer without air gaps, impurities, and voids. The magnified emission patterns shown in Fig. 6c clearly define all four pixels with sharp edges. This strongly suggests that the process used to transfer the EGBS onto the bank structure realizes a contact that prevents the interface being separated. In addition, If the physical contact between graphene/organic layers fail, there will be no emission at all, dead pixels. The absence of physical gap at the interface is achieved by laminating backside graphene layer on organic layers. In Fig. 6c, all the pixels are fully luminescent, but their luminance is microscopically different. The inhomogeneous emission pattern may not be responsible for the poor physical contact, but for irregular thickness of graphene layer. The graphene layer used in this study is synthesized on a polycrystalline Ni substrate using chemical vapor deposition. The variation of local thickness of graphene layer is expected to incur luminescence unevenness through a two pathways; local carrier injection and light transmittance. In addition, the fabricated device with MLG top electrode shows stable luminance characteristics at high luminance. If the EGBS is not successfully laminated on the organic layer, high luminance cannot be achieved because of device damage from high electric field. Improving the thickness uniformity of CVD-grown MLG will be a subject of future study.

We also fabricated a large OLED segment panel with a graphene top electrode, as shown in Fig. 6d. The laminated graphene electrode had dimensions of approximately 23 mm × 23 mm. The “ETRI” logo is shown and the panel uniformly emits a yellowish-green light. A motion picture of the segment panel is provided in Supplementary Video 2. This result indicates that the MLG top electrode has the potential to replace the currently used metal top electrodes for use in large optoelectronic and energy harvesting devices.

## Discussion

To date, various efforts have been made towards the realization of OLEDs integrated on a thin-film-transistor (TFT) backplane with pixels separated by a bank pattern, so as to enhance the performance of the STM top electrode. To this end, various materials have been studied, including TCOs, metal nanowires, CNTs, and graphene. In this work, we demonstrate a novel lamination process through which graphene is employed as a TCTE for use in OLEDs. Our approach enables residue-free transfer, flexion bonding, and conformal contact with the MLG. In addition, a low-temperature dry fabrication process is developed. Furthermore, all of these techniques are adaptable to the current OLED fabrication process. The overall performance of the G-TOLED fabricated using our lamination process is comparable to that of Ag-TOLEDs, because of the superior optical properties of MLG in spite of high sheet resistance of MLG. The developed bonding technology using MLG as an STM alternative has potential application in a wide range of optoelectronic devices, such as energy-harvesting devices, TFTs, and light sensing devices, as well as light-emitting devices. Furthermore, we believe that our new bonding method can also be applied to various 2D layered materials such as MoS_2_, WSe_2_, and MoTe_2_.

## Methods

### EGBS materials

The developed bonding structure is composed of a MLG/BL/PET/PDMS structure. We used MLG synthesized on a Si WF/Ni substrate via chemical vapor deposition. We purchased methacryloxypropyl terminated polydimethylsiloxanes (DMS-R22, Gelest Inc.) as a BL and 25-μm-thick PET film (H34P (F), Kolon Inc.) as a supporting layer, which contains an acrylate adhesive on one face and a urethane adhesive on the other. A two-component PDMS (SYLGARD^®^ 184, Dow Corning Co.) was heated at 80 °C for 1 h with a 10:1 base-to-curing-agent mixing ratio, following degassing for approximately 1 h at a pressure of approximately 5 × 10^−2^ Torr. A FeCl_3_ solution (CE-100, Transene Company. Inc.) was used as a Ni etchant, and a HCl solution of 37% concentration (Sigma-Aldrich Co.) was used to clean the MLG after the Ni etching.

### EGBS fabrication

The BL/PET bonding structure was prepared as follows. The bonding agent was prepared by mixing DMS-R22 with 2-hydroxy-2-methyl-1-phenyl-propan-1-one (Ciba® DAROCURAE®1173, Ciba Specialty Chemicals Inc.) of 1.5 wt% as a photoinitiator. Liquid bridging was achieved via UV curing of a bonding agent, which was added between the acrylate adhesive face of the PET film and the SAM-treated Si WF. A heptadecafluoro-1,1,2,2-tetrahydrodecyl)trichlorosilane (Gelest Inc.) SAM material was used for the treatment (see Supplementary Information 1 online). Then, the BL/PET was obtained after the physical detachment from the Si WF/SAM/BL/PET. The EGBS was synthesized via the three fabrication processes: gapless contact; Ni metal etching; and MLG cleaning. First, the BL of the BL/PET was attached to the MLG on the Si WF/Ni/MLG via conformal and impurity-free contact, which was inosculated by dispersive adhesion without air gaps. Then, the Ni metal was etched using a FeCl_3_ solution (1:50 ratio for FeCl_3_ solution: distilled water). When the Ni etching process was complete, the Si WF was separated from the bottom of the Si WF/Ni/MLG/BL/PET and an EGBS was fabricated. The fabricated EGBS was cleaned by rinsing five times in running DI water to remove the residual etchant. Next, the graphene bonding stack was dipped in the diluted HCl solution (1:10 ratio for HCl:H_2_O) for 10 min, to remove any residual Fe^3+^ ions. Finally, the sample was washed with distilled water to remove any residual HCl solution and then dried for 1 h at 60 °C with running nitrogen gas (N_2_) at a pressure of 1 × 10^−2^ Torr. The EGBS/PDMS sample was fabricated by establishing contact between the EGBS and the PDMS layer.

### Device fabrication

The inverted G-TOLEDs were fabricated with the following structure: glass/ITO/Li-doped TRE/TRE/(Ir(ppy)_2_(m-bppy))-doped PGH02/TcTa/TAPC/HAT-CN/MLG/BL/PET. Additionally, to compare the device performance, we fabricated a reference device containing a thin-Ag (20 nm) top electrode in place of the MLG/BL/PET. All of the organic and Ag layers comprising the TOLED were sequentially vacuum-deposited *in situ* via thermal evaporation on patterned ITO-coated glass substrates at a base pressure of less than 5 × 10^−7^ Torr. A pixel was defined using a bank structure with a thickness of approximately 1 μm. The emissive active area of the fabricated devices was 1.5 × 1.5 mm^2^. The bonding structure of the EGBS/PDMS was transferred to the glass/cathode/EIL/ETL/EML/HTL/HIL stack via lamination at 60 °C under a pressure of approximately 110 kPa. Finally, the G-TOLED was completed by physically detaching the PDMS from the TOLED with the EGBS/PDMS graphene bonding structure. The fabricated OLEDs were transferred to an inert environment glove-box, where they were encapsulated using a UV-curable epoxy and a glass cap containing a moisture absorbent.

### Device Characterization

The transmittance and reflectance of the OLEDs with different top electrodes were characterized using an ultraviolet-visible-near-infrared (UV-Vis-NIR) spectrophotometer (LAMBDA 750, PerkinElmer). The J-V characteristics of the devices were measured using a Keithley-238 source-measure unit, and the luminance and EL spectra were examined using a goniometer-equipped spectroradiometer (CS-2000, Konica Minolta). The efficiencies of the OLEDs were calculated from the I-V-L characteristics, their EL spectra, and the angular distributions of the EL intensities. All measurements were conducted at room temperature.

### TOLED simulation

To calculate the light distribution and intensity of each TOLED as delivered to an infinitely thick substrate medium, we used custom Matlab codes following an advanced classical coherent electromagnetic theory, as summarized by Furno *et al.*^36^. This program was based on multilayer thin-film optics. The main input variables were the reflective index (*n*), extinction coefficient (*k*), and film thickness. To obtain realistic simulation results, we used all of the measured *n* and *k* values of the organic materials, as determined using an elipsometer (M-2000D, J.A. Woollam Co.). The *n* and *k* values of silver and graphene are given in the literature^41^,^42^.

## Additional Information

**How to cite this article**: Tae Lim, J. *et al.* Flexion bonding transfer of multilayered graphene as a top electrode in transparent organic light-emitting diodes. *Sci. Rep.* **5**, 17748; doi: 10.1038/srep17748 (2015).

## Supplementary Material

Supplementary Information

Supplementary Video 1

Supplementary Video 2

## Figures and Tables

**Table 1:** Simulated fraction of emitted optical power into the various modes supported by the Ag- and G-TOLEDs structures.

Device	Out		Substrate		Waveguided	SPP	Absorption
	Bottom	Top	Glass	BL			
Ag-TOLED	8.97	3.06	30.57	–	33.22	16.24	7.94
G-TOLED	7.94	7.83	14.73	8.54	46.46	6.60	7.90
