# Supplementary material for: Flexion bonding transfer of multilayered graphene as a top electrode in transparent organic light-emitting diodes
Source: Sci Rep. 2015 Dec 2;5:17748. doi: 10.1038/srep17748 (PMC4667220; doi:10.1038/srep17748)
Supplement: Supplementary Information [file srep17748-s1.doc]

**SUPPLEMENTARY INFORMATION**

**Flexion bonding transfer of multilayered graphene as a top electrode in transparent organic light-emitting diodes**

Jong Tae Lim, Hyunkoo Lee, Hyunsu Cho, Byoung-Hwa Kwon, Nam Sung Cho, Bong Kuk Lee, Jonghyurk Park, Jae Su Kim, Jun-Han Han, Jong-Heon Yang, Byoung-Gon Yu, Chi-Sun Hwang, Seong Chu Lim & Jeong-Ik Lee

The supplementary information includes seven figures and two videos.

**1. Fabrication of the hydrophobic self-assembly** **monolayer**

A silicon wafer (Si WF) with hydroxide terminations was obtained via ultraviolet ozone (UVO) treatment for 10 min during step 1 in Fig. S1. In step 2 in Fig. S1, the UVO-treated Si WF layers in a container were blocked from air, and a few drops of the self-assembly monolayer (SAM) material, (heptadecafluoro-1,1,2,2-tetrahydrodecyl)trichlorosilane (Gelest Inc. CAS No.: 78560-44-8), was added near to the Si WF. This container was heated to 130°C for 1 h for full coverage of the SAM on the silicon wafer. The SAM is covalently linked to functional groups on the Si WF and is therefore highly stable. After heating, the container was cooled to room temperature. Then, the Si WF/SAM sample was cleaned for 5 min under sonication in toluene and was heated for 30 min at 110°C on a hot plate. In step 3 in Fig. S1, the fabricated Si WF/SAM sample was sequentially cleaned twice via ultrasonication using toluene, acetone, methanol, and distilled water, and it was further dried in 80 ^o^C for 30 min.


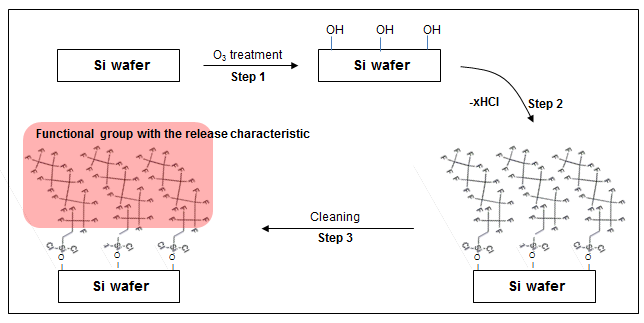


**Figure S1.** Fabrication process of a Si WF/SAM sample.

**2. Surface roughness of the Si and SAM-treated Si wafer**

Topographic images of all samples were obtained using atomic force microscope (Dimension 3100 with a Nanoscope controller, Veeco) operating in a tapping mode with a tip (Nanosensors Inc.) at a resonance frequency of approximately 250 kHz. The scan speed was set to 0.5 Hz per line, and the images were taken over a 5×5 μm^2^ region. The RMS surface roughness of the Si wafer in Fig. S2a and SAM-treated Si wafer in Fig. S2b, over an area of 5×5 μm^2^ were 0.23 and 0.36 nm, respectively.


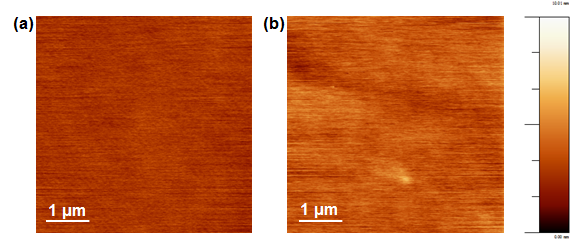


**Figure S2.** Topographic images of (a) a Si WF and (b) a Si WF/SAM. The maximum value of the height scale bar is 10 nm.

**3. Polarized Raman spectroscopy of Ni/MLG and MLG/BL/PET**

Raman spectra were collected by a confocal Raman microscopy (NTEGRA Spectra, NT-MDT) system with an excitation laser-wavelength of 532 nm and a 100X objective (NA = 0.9). The instrumental spectral resolution was 1.0 cm^-1^. The polarization of incident laser was controlled with a half-wave plate. The angle was increased by 10^o^ and the Raman spectra from 550 ~ 2900 cm^-1^ was recorded.

**Figure S3.** The intensity variation of (a) G^-^ and (b) G^+^ peaks of Ni/MLG as a function of polarization angle of the incident laser. The intensity variation of (c) G^-^ and (d) G^+^ peaks of MLG/BL/PET as a function of polarization angle of the incident laser. (e) Raman spectra of BL/PET and MLG/BL/PET.

**4. Wide scan x-ray photoelectron spectroscopy**

We carried out a wide scan XPS at 650 eV. When MLG is separated from Si/Ni/MLG through chemical etching using a FeCl_3_ solution, the etching process may remain salt of metal chloride. However, wide scan XPS did not detect any related element such as Cl 2p (199 eV), Cl 2s (271 eV), Ni 3p (67 eV), Fe 3p (53 eV), which is shown in a figure below. This result strongly supports that there are no impurities at the interface of EGBS and TOLED. Such a clean interface is essential for conformal contact in our device.

**Figure S4**. Wide scan XPS at 650 eV on the EGBS

**5. Reproducibility of G-TOLEDs in Luminance current efficiency (LCE)-luminance (L) characteristics**

LCE-L characteristics of 8 different devices has been monitored as shown in Fig. S5. In the figure, all our G-TOLEDs successfully emit. And, the LCE of top and bottom emission is highly consistent among different device in addition to the similar trend in the ratio of top to bottom emission. This result implies that our flexion transfer techniques is highly reproducible.


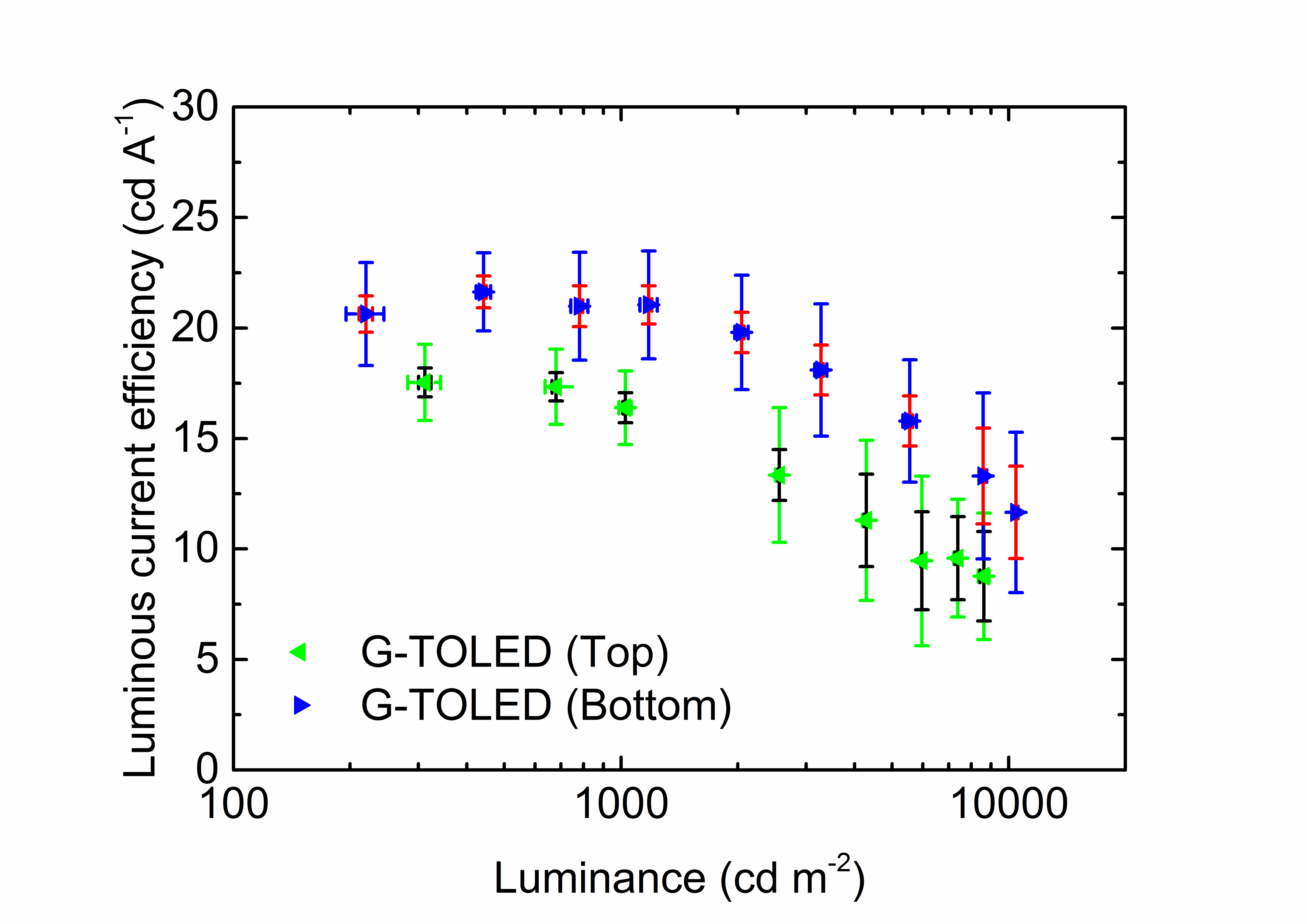


**Figure S5**. LCE-L characteristics with standard deviation (blue and green line) and standard error (red and black line) of G-TOLED

**6. Simulated the power dissipation spectra of Ag- and G-TOLED at 550 nm.**


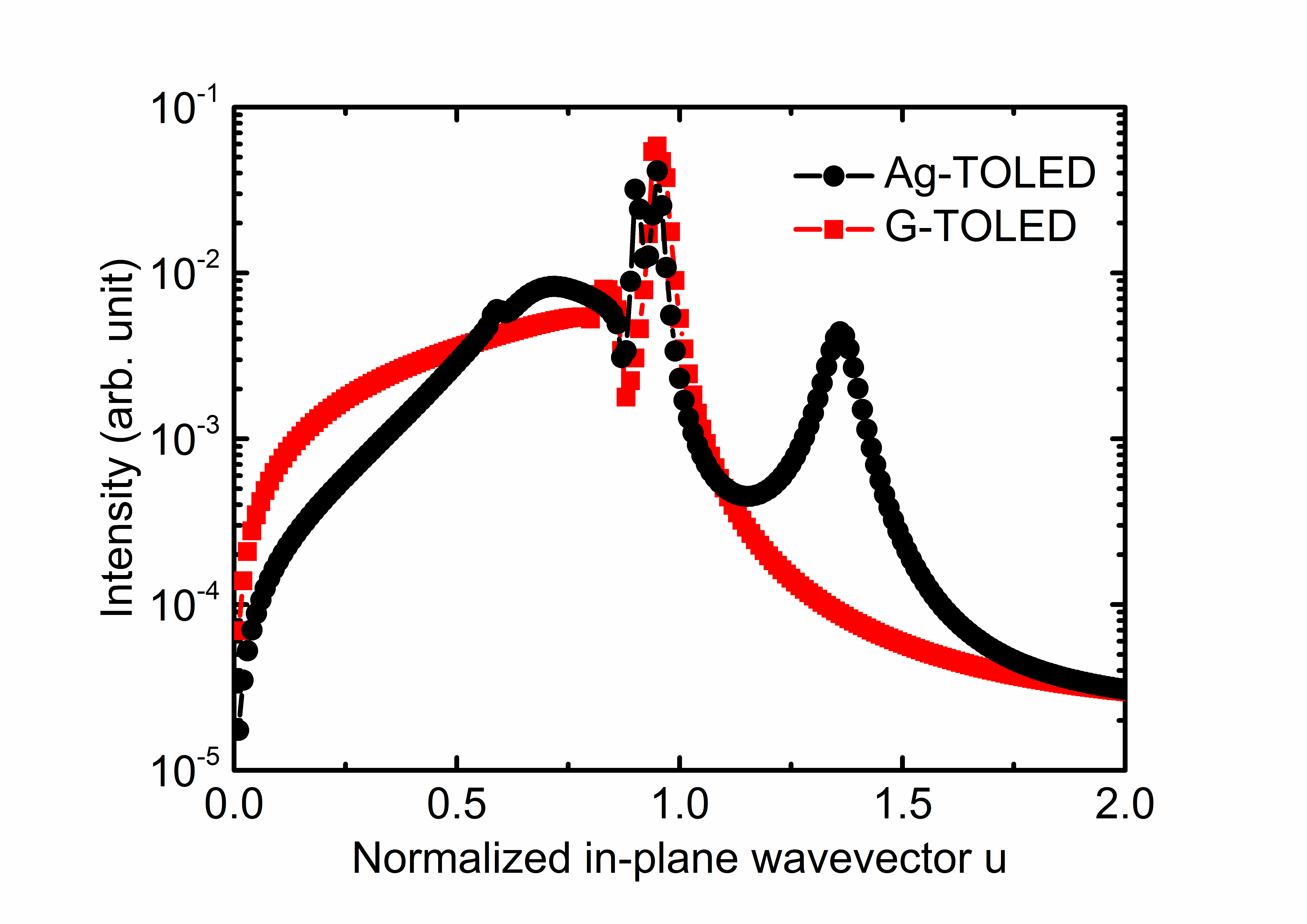


Figure S6. Simulated power dissipation spectra of Ag- and G-TOLED at 550 nm.

**7. Angular distribution of EL intensity and spectra of Ag- and G-TOLED.**

**
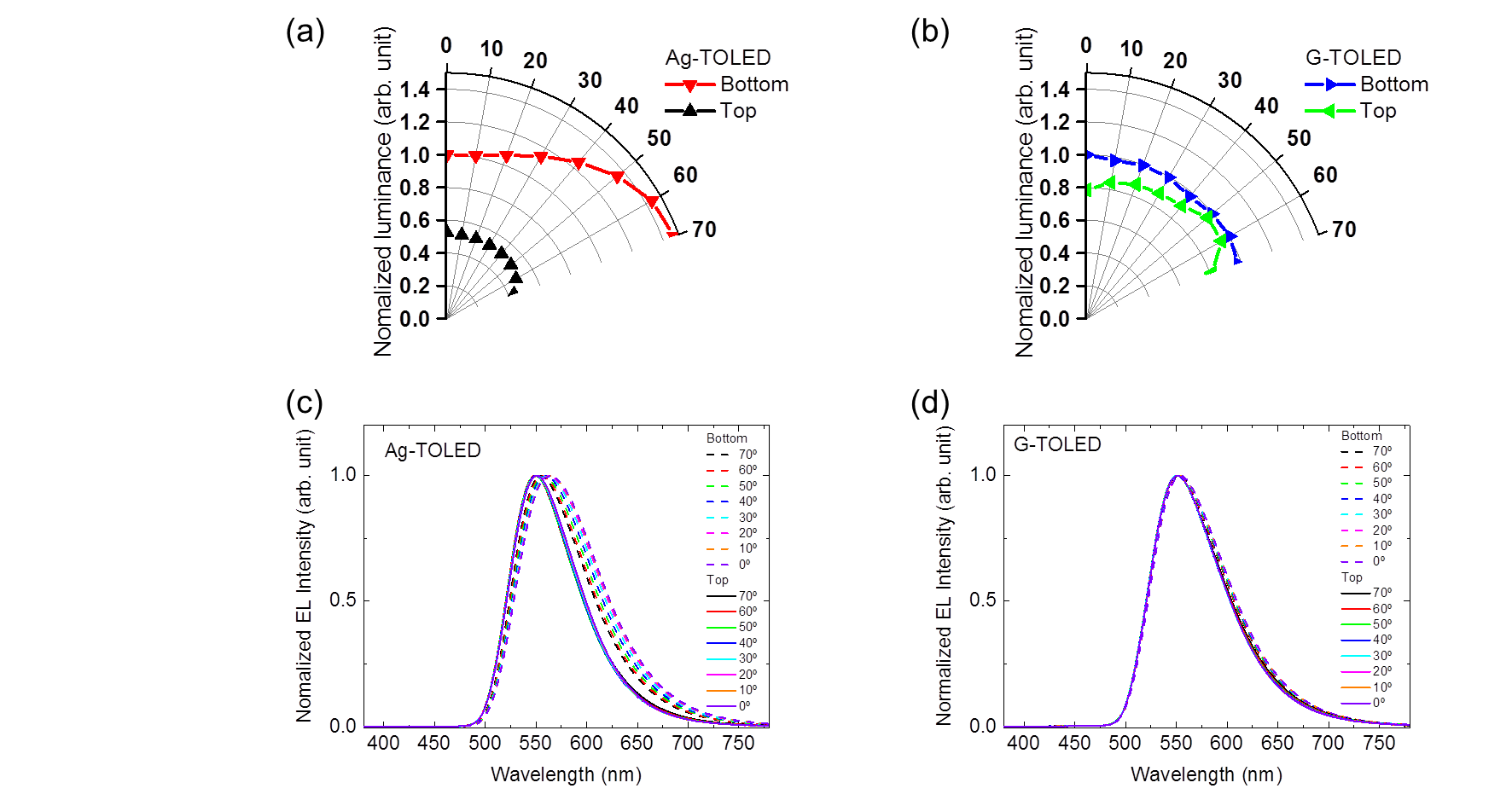
**

Figure S7. Angular dependence of emissin characteristics of TOLEDs. (a) and (b) Angular distribution of EL intensity of Ag- and G-TOLED, respectively. (c) and (d) EL spectra depending on viewing angles for Ag- and G-TOLED, respectively.

**Supplementary Video**

**Supporting Video 1.** The movie captures an emission from the four pixels of the yellowish transparent OLED with an active area of 1.5×1.5 mm^2^. The transparent OLED is simultaneously luminescent through both the top and bottom surfaces. Click the play button to start the animation.

**Supporting Video 2**. The movie records emissions from the segment-type pixels of the yellowish transparent OLED with MLG area of 23×23 mm^2^. Click the play button to start the animation.
